# Supplementary material for: Joint analysis of phenotype-effect-generation identifies loci associated with grain quality traits in rice hybrids
Source: Nat Commun. 2023 Jul 4;14:3930. doi: 10.1038/s41467-023-39534-x (PMC10319794; doi:10.1038/s41467-023-39534-x)
Supplement: Supplementary file 1 — Supplementary information [file 41467_2023_39534_MOESM1_ESM.pdf]

Joint analysis of phenotype-effect-generation identifies loci associated with grain quality traits in rice hybrids

Li *et al.*

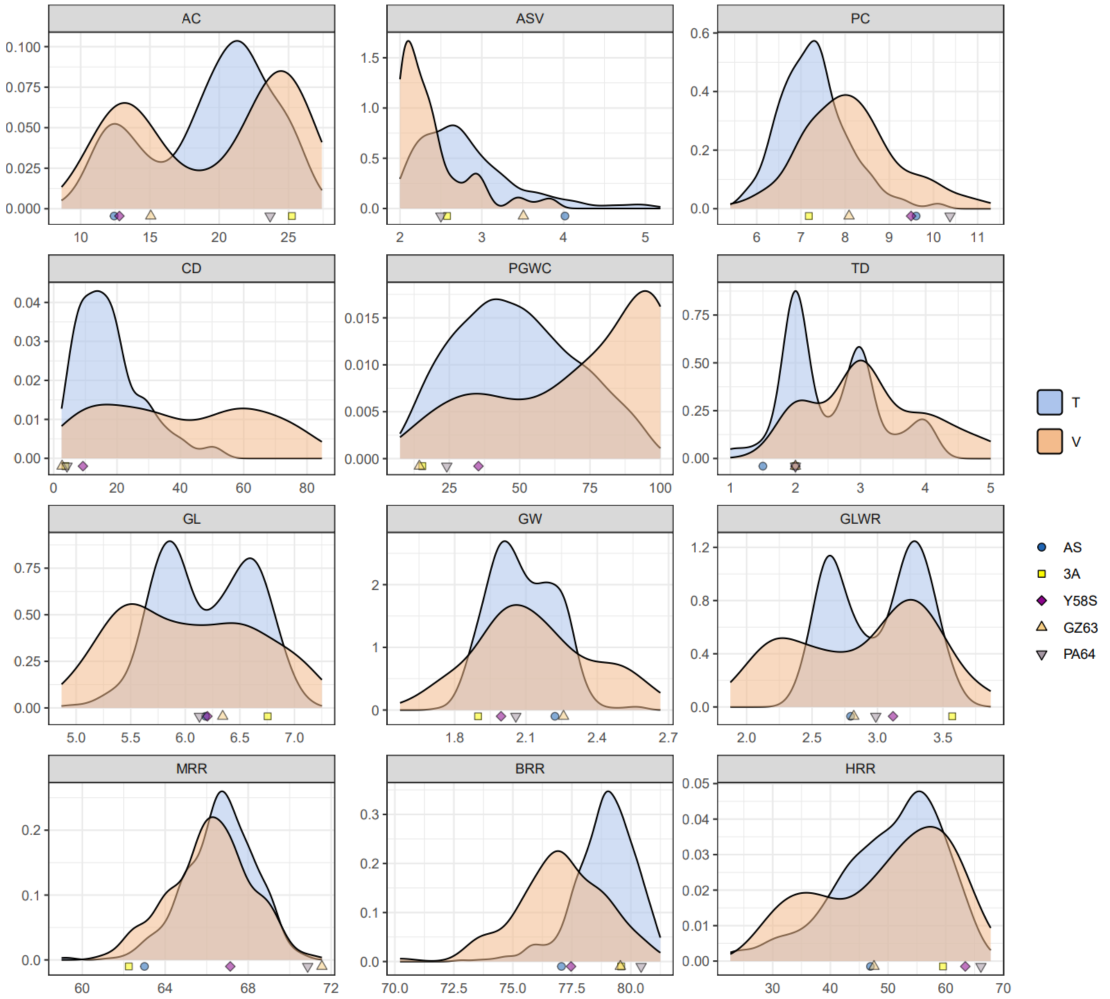

Supplementary Figure 1. Phenotypic distributions of 12 rice quality traits in parental inbred varieties and hybrid testcrosses. The distributions of the 12 traits are illustrated as density plots for inbred varieties (V) and hybrid testcrosses (T). The vertical axis represents the frequency density of observations. The horizontal axis shows the observations. The positions of the five female varieties are indicated by different symbols. Source data are provided as a Source Data file.

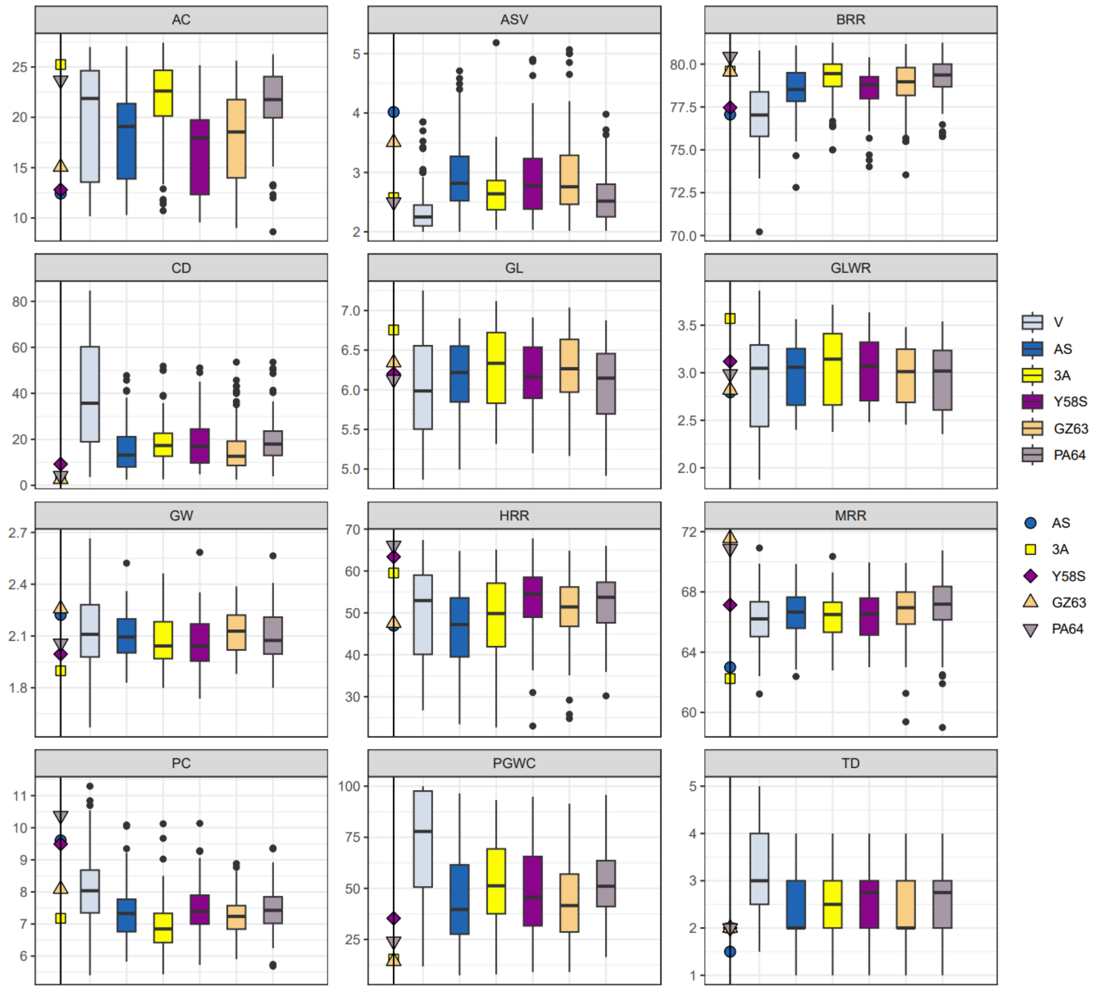

Supplementary Figure 2. Phenotypic distributions of 12 rice quality traits in parental inbred varieties and sub-hybrid testcrosses. The distributions of the 12 traits are illustrated as box plots for inbred varieties (V) and five hybrid testcross subpopulations with different female parents. The vertical axis is the observations. The horizontal edges of the boxes are the lower and upper quartiles. The horizontal lines are the medians. The whiskers and black dots indicate the ranges of the distributions. The positions of the five female parents are indicated by different symbols on the vertical straight lines. Source data are provided as a Source Data file.

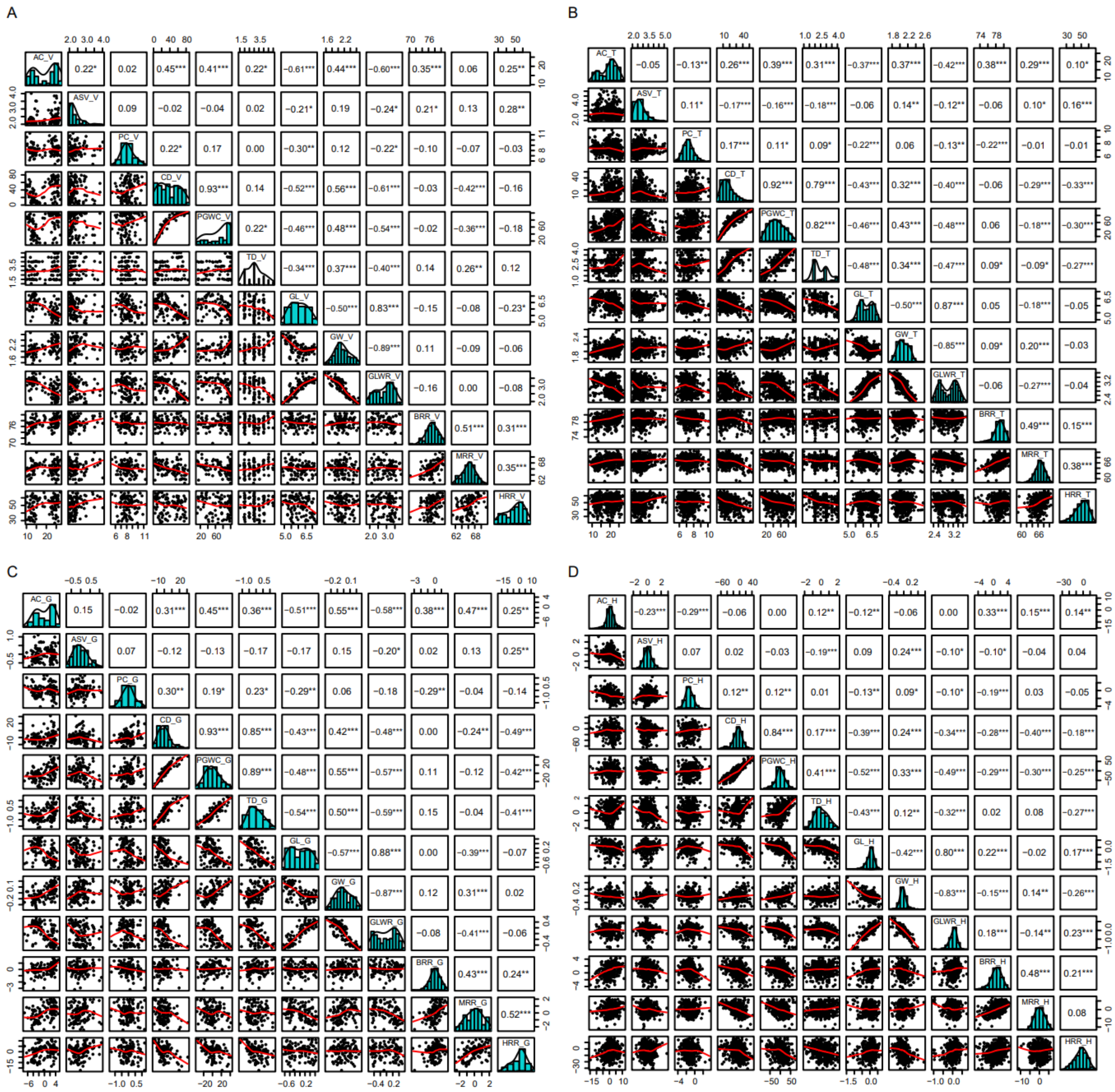

Supplementary Figure 3. Distribution and correlations among 12 traits in four types of phenotypes. The distributions are illustrated as histograms on the diagonals for each trait on inbred variety observations (A), hybrid testcross observations (B), general combine ability (C), and heterosis (D). Within each type of phenotype, correlation and scatter plots are illustrated above the diagonals and below the diagonals, respectively. Source data are provided as a Source Data file.

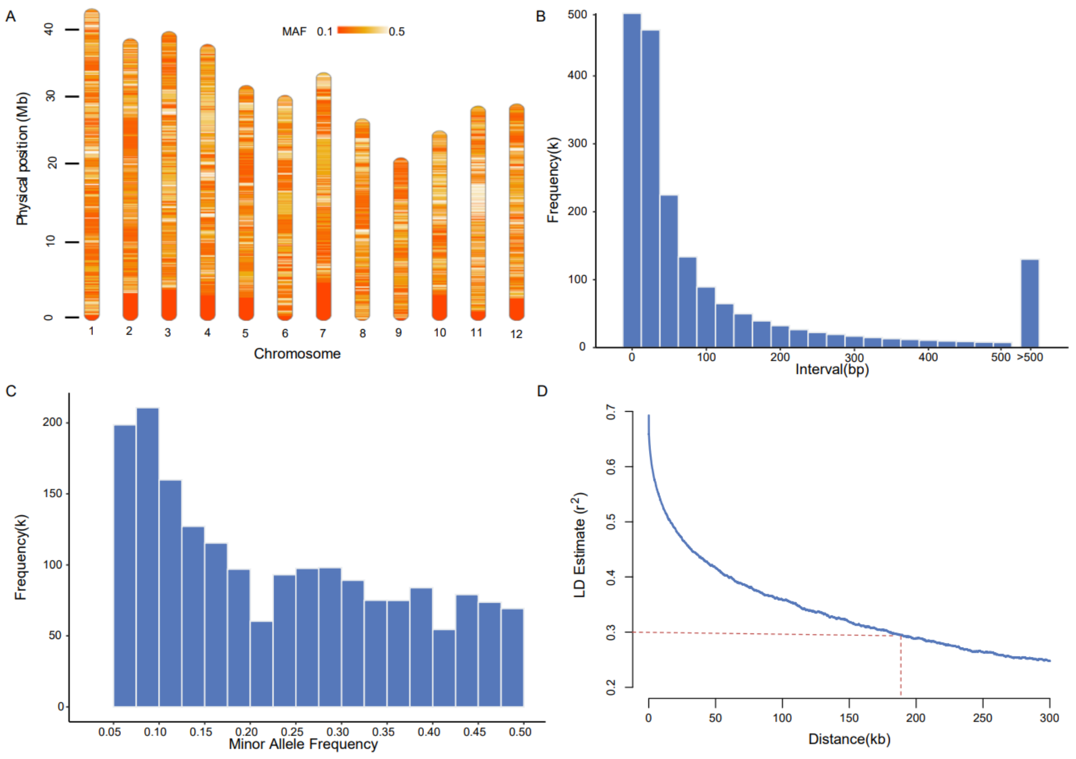

Supplementary Figure 4. Properties of single nucleotide polymorphisms (SNPs). A total of 7,734,465 raw SNPs were obtained from 118 parental varieties genotyped by whole genome sequencing with the Illumina HiSeq2500 platform. The distribution of these SNPs on the rice genome is illustrated chromosome-wise with a heatmap indicating minor allele frequency (MAF) (A). Among all the raw SNPs, 1,619,588 SNPs passed filters and quality control (missing rate <20%, MAF>5%). The marker density of the filtered SNPs is displayed by a histogram according to the interval of adjacent SNPs (B). Among all intervals, 95% are less than 200 kb. The distribution of MAFs is detailed as a histogram for filtered SNPs (C). Linkage disequilibrium (LD) decay is displayed as the R square between markers with different distances (D). The R square is above 0.3 on average for two markers with a distance of less than 200 kb. Source data are provided as a Source Data file.

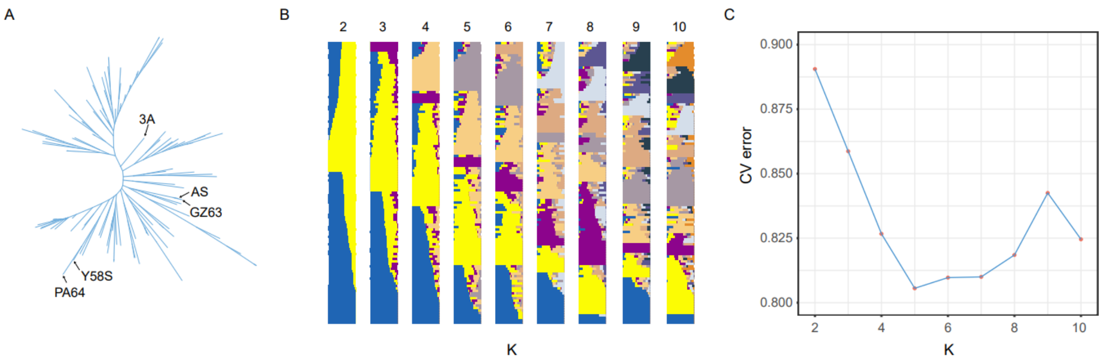

Supplementary Figure 5. Population structure analyses among the 120 parental inbreds. The five female parents have substantial coverage of genetic diversity among the 120 parental varieties (115 are male parents), as demonstrated in the neighbour-joining tree developed by using the 1,619,588 filtered SNPs (A). Population structure was analyzed using Admixture software (3.0) for K=2 to 10. The parental inbreds are displayed in the order of the population components with K=2 (B). Optimization of cross-validation (CV) was performed over K from 2 to 10. The cross-validation error reached the minimum when K=5 (C). Source data are provided as a Source Data file.

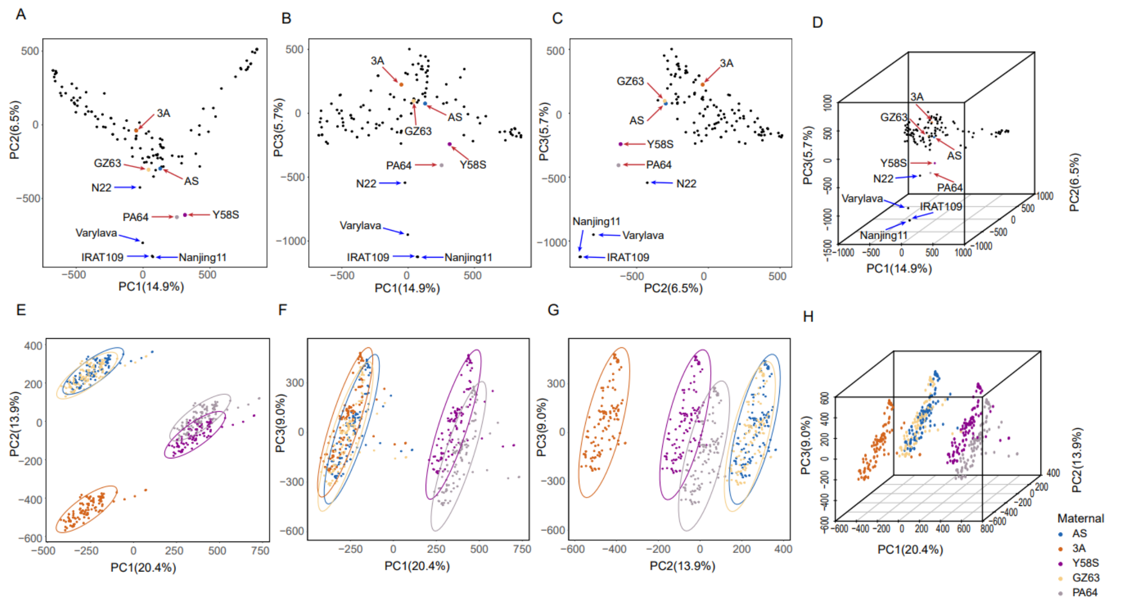

Supplementary Figure 6. Population structure among parental inbreds and hybrids revealed by principal component analysis. Principal component analysis was conducted on the 1,619,588 filtered SNPs among inbreds (A to D on the upper panel) and hybrids (E to H on the lower panel). The first three principal components (PCs) of inbred varieties are illustrated pairwise between PC1 and PC2 (A), PC1 and PC3 (B), and PC2 and PC3 (C) and in 3D format (D). The five female parents are labelled in colours and shown by arrows. Similarly, the PCs of hybrids are illustrated pairwise between PC1 and PC2 (E), PC1 and PC3 (F), and PC2 and PC3 (G) and in 3D format (H). The hybrids were categorized by their five female parents. The colours of the parents (A to D) are the same as those of their hybrids (E to H). The red arrows label the female parents and the blue arow label the female parent outliers. Source data are provided as a Source Data file.

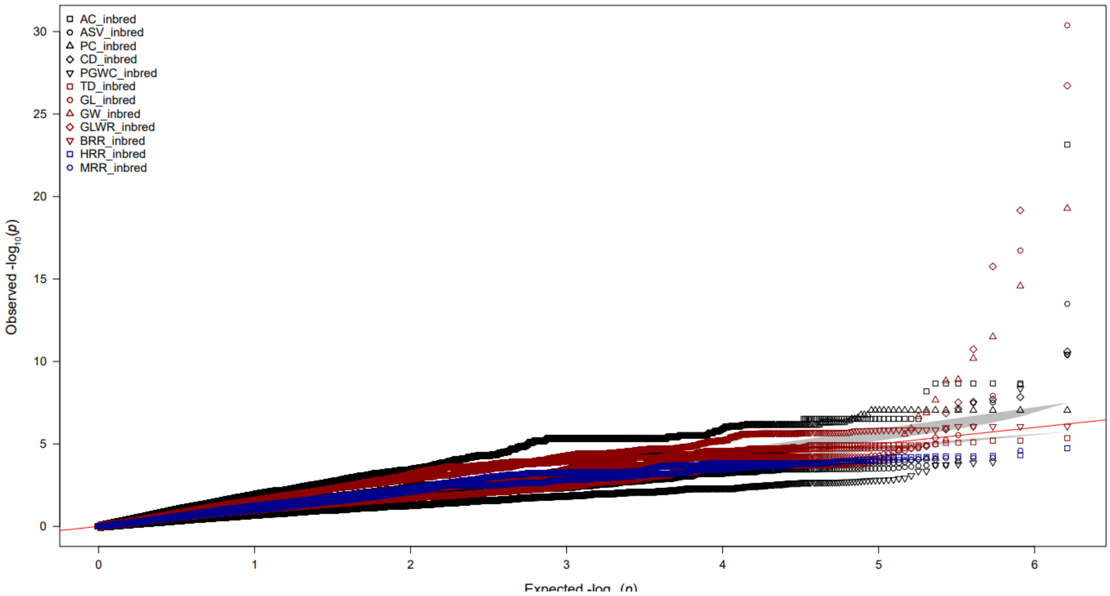

Supplementary Figure 7. Multiple QQ plot for 12 traits in the inbred population. Source data are provided as a Source Data file.

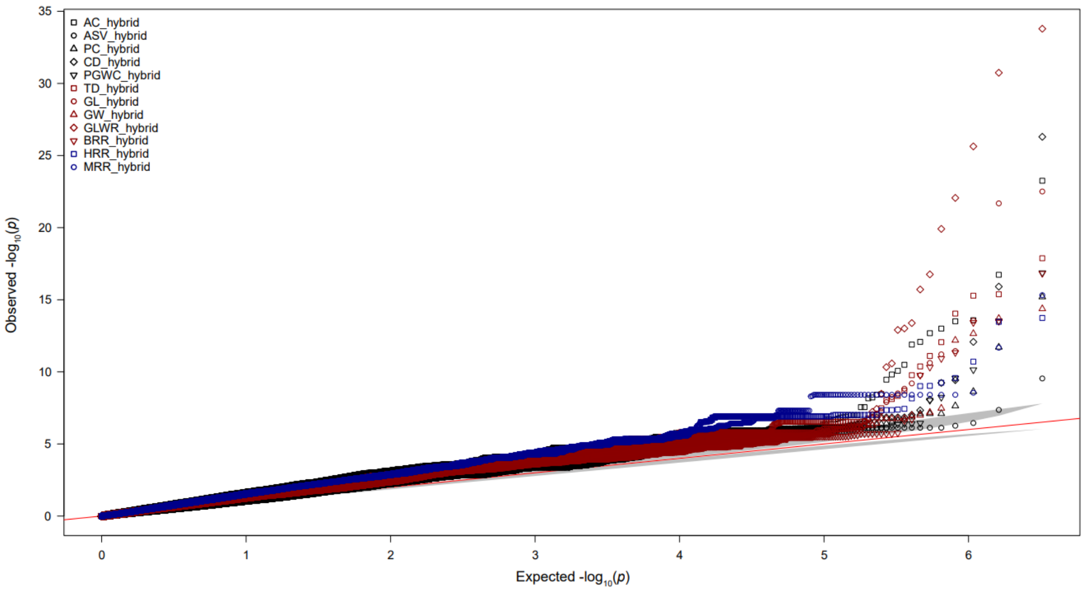

Supplementary Figure 8. Multiple QQ plot for 12 traits in the hybrid population. Source data are provided as a Source Data file.

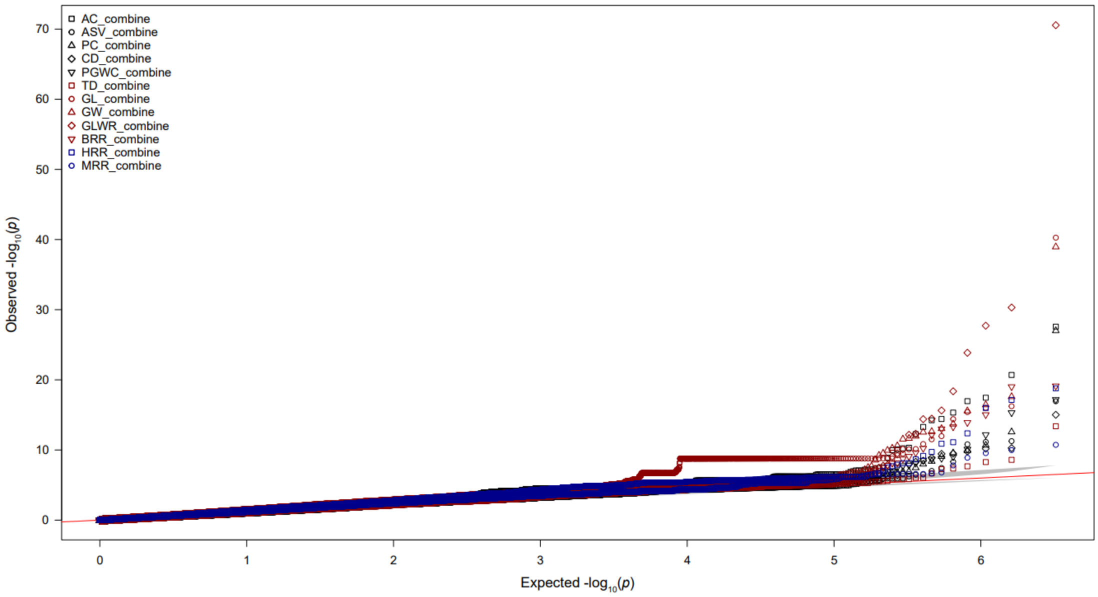

Supplementary Figure 9. Multiple QQ plot for 12 traits in the combine population. Source data are provided as a Source Data file.

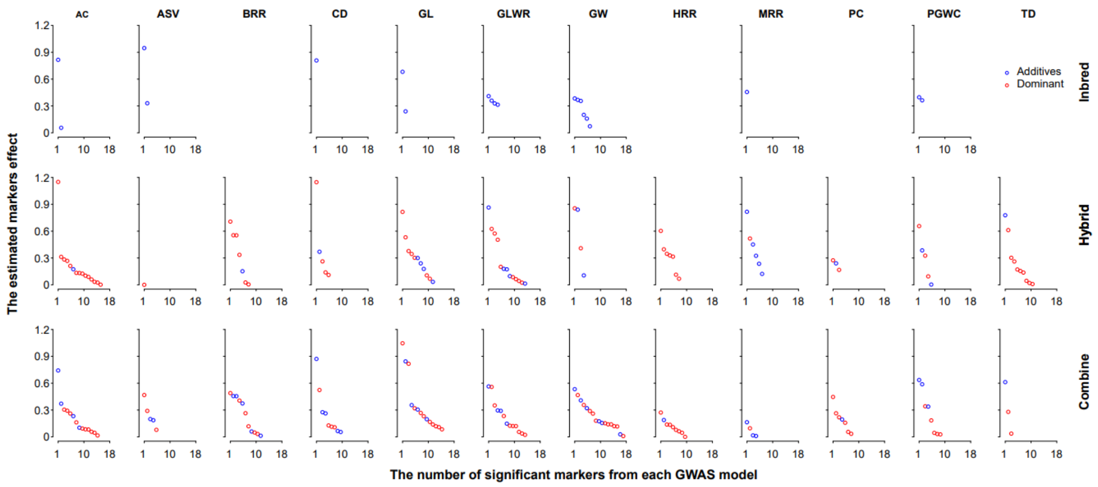

Supplementary Figure 10. The additive and dominant genetic effects of the associated markers. The association analyses were conducted on standardized phenotypes with zero mean and standard deviation of one. The absolute values are displayed for the additive genetic effects (red) and dominant effect (blue). Markers are arranged horizontally in decreasing order of effects within traits and the three types of analyses using inbreds, hybrids, and combined. Source data are provided as a Source Data file.

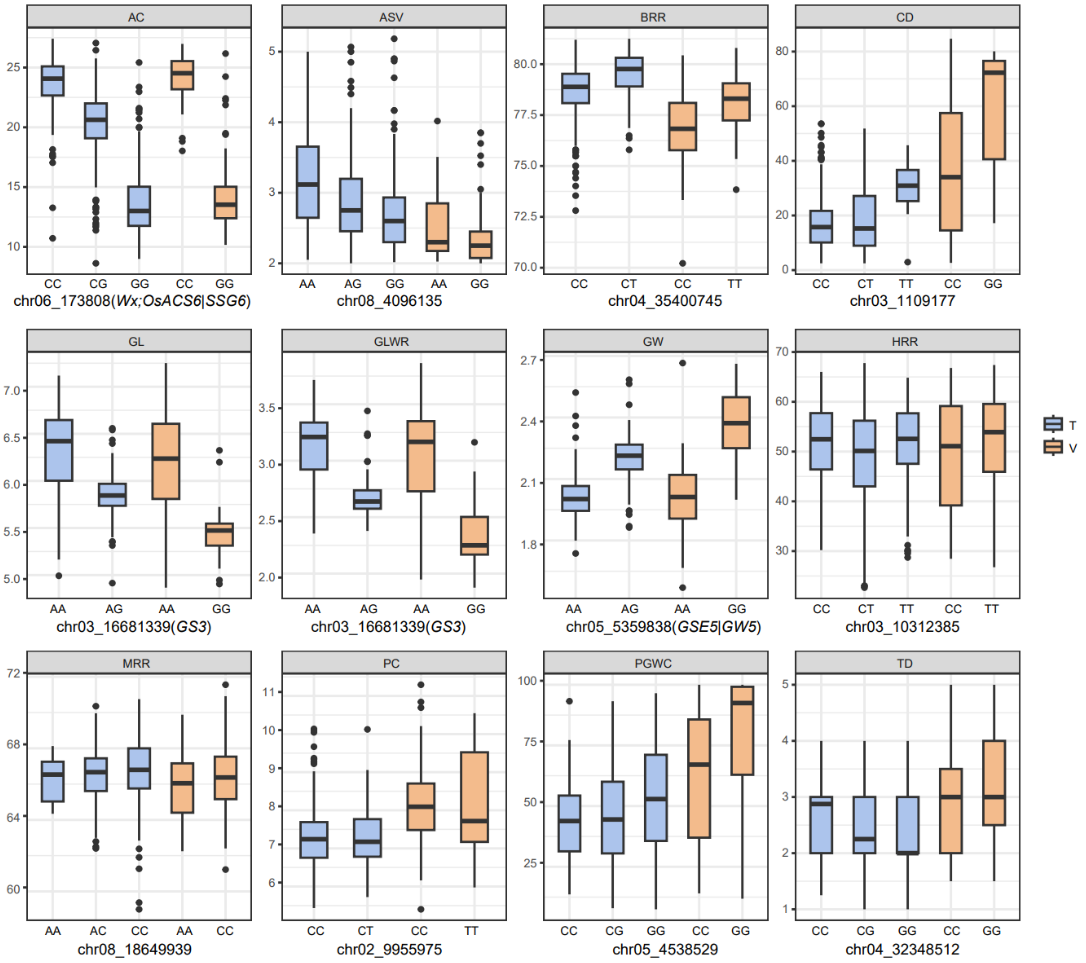

Supplementary Figure 11. Phenotype distribution withing genotypes of the most associated SNP for each trait. The distribution is displayed as Box plot for inbred varieties (V) and testcross (T) separately. The most associated SNPs were selected from either additive or dominant genetic effects across three analyses (inbred, hybrid and combination). Three know genes are included in the selection (*GS3*, *GW5* and *Wx)*. The sample size of each boxplot in this figure are different. Source data are provided as a Source Data file.
