# Supplementary material for: Joint analysis of phenotype-effect-generation identifies loci associated with grain quality traits in rice hybrids
Source: Nat Commun. 2023 Jul 4;14:3930. doi: 10.1038/s41467-023-39534-x (PMC10319794; doi:10.1038/s41467-023-39534-x)
Supplement: Supplementary file 3 — Description of Additional Supplementary Files [file 41467_2023_39534_MOESM3_ESM.docx]

**Description of Additional Supplementary Files**

File Name: Supplementary Data 1

Description: Associated SNPs and candidate genes information

File Name: Supplementary Data 2

Description: Names and origins of the 115 indica rice accessions and 5 male sterile lines

File Name: Supplementary Data 3

Description: Phenotype values of inbred varieties(*V*)

File Name: Supplementary Data 4

Description: Phenotype values of hybrid testcrosses (*T*)

File Name: Supplementary Data 5 in zip format

Description: Genotype data of parental lines

File Name: Supplementary Data 6

Description: General combine ability of inbred varieties(*G*)

File Name: Supplementary Data 7

Description: Heterosis of hybrid testcrosses (*H*)
