# Supplementary material for: Joint analysis of phenotype-effect-generation identifies loci associated with grain quality traits in rice hybrids
Source: Nat Commun. 2023 Jul 4;14:3930. doi: 10.1038/s41467-023-39534-x (PMC10319794; doi:10.1038/s41467-023-39534-x)
Supplement: Supplementary file 11 — Reporting Summary [file 41467_2023_39534_MOESM11_ESM.pdf]

Corresponding author(s): NCOMMS-22-14948

Last updated by author(s): May 17, 2023

Reporting Summary

Nature Portfolio wishes to improve the reproducibility of the work that we publish. This form provides structure for consistency and transparency in reporting. For further information on Nature Portfolio policies, see our Editorial Policies and the Editorial Policy Checklist.

Statistics

For all statistical analyses, confirm that the following items are present in the figure legend, table legend, main text, or Methods section.

n/a	Confirmed	
☐	☒	The exact sample size (*n*) for each experimental group/condition, given as a discrete number and unit of measurement
☐	☒	A statement on whether measurements were taken from distinct samples or whether the same sample was measured repeatedly
☐	☒	The statistical test(s) used AND whether they are one- or two-sided<br>*Only common tests should be described solely by name; describe more complex techniques in the Methods section.*
☐	☒	A description of all covariates tested
☐	☒	A description of any assumptions or corrections, such as tests of normality and adjustment for multiple comparisons
☐	☒	A full description of the statistical parameters including central tendency (e.g. means) or other basic estimates (e.g. regression coefficient) AND variation (e.g. standard deviation) or associated estimates of uncertainty (e.g. confidence intervals)
☒	☐	For null hypothesis testing, the test statistic (e.g. *F*, *t*, *r*) with confidence intervals, effect sizes, degrees of freedom and *P* value noted<br>*Give P values as exact values whenever suitable.*
☒	☐	For Bayesian analysis, information on the choice of priors and Markov chain Monte Carlo settings
☒	☐	For hierarchical and complex designs, identification of the appropriate level for tests and full reporting of outcomes
☐	☒	Estimates of effect sizes (e.g. Cohen's *d*, Pearson's *r*), indicating how they were calculated

Our web collection on statistics for biologists contains articles on many of the points above.

Software and code

Policy information about availability of computer code

Data collection	The four types of observations (V, T, G, and H) were normalized within each type to eliminate the difference between scales and averages. They were staged together as a single phenotype vector (Y) in the combined analysis. Their corresponding genotype matrix was staged correspondingly. V and G share the same genotype matrix, while T and H share the same genotype matrix. Homozygous genotypes are coded as 0 and 2, while heterozygous genotypes are coded as 1 in the additive genotype matrix (A). Similarly, both homozygous genotypes are coded as 0, while the heterozygous genotype is coded as 1 in the dominant genotype matrix (D). Note that the dominant genotypes are all 0s for V and G.
Data analysis	The additive genotype matrix and dominant genotype matrix were staged together (left and right) for GWAS with the BLINK multiple loci model implemented in GAPIT (version 3.0, Wang, J. & Zhang, Z. GAPIT Version 3: Boosting Power and Accuracy for Genomic Association and Prediction. Genomics. Proteomics Bioinformatics 19, 1–12 (2021)). At the end of the iterations for testing additive and dominant marker effects, the associated marker effects, either additive or dominant, were selected as covariates to test the model. We named this pipeline the joint analysis of phenotypes, effects, and generations (JPEG). The additional fixed effect covariates included the first three principal components derived from the additive genotypes of all SNPs and the dummy variables of female parents for the testcross. The association was determined with the threshold of 1% type I error after Bonferroni multiple test correction on both additive and dominant markers.

For manuscripts utilizing custom algorithms or software that are central to the research but not yet described in published literature, software must be made available to editors and reviewers. We strongly encourage code deposition in a community repository (e.g. GitHub). See the Nature Portfolio guidelines for submitting code & software for further information.

Data

Policy information about availability of data

All manuscripts must include a data availability statement. This statement should provide the following information, where applicable:

- Accession codes, unique identifiers, or web links for publicly available datasets
- A description of any restrictions on data availability
- For clinical datasets or third party data, please ensure that the statement adheres to our policy

The raw DNA sequencing data of 120 rice inbred lines analysed in the current study are available in the NCBI Sequence Read Archive, https://www.ncbi.nlm.nih.gov/sra/?term=SRP080763 and https://www.ncbi.nlm.nih.gov/sra/?term=SRP080834.

Field-specific reporting

Please select the one below that is the best fit for your research. If you are not sure, read the appropriate sections before making your selection.

- [x] Life sciences
- [ ] Behavioural & social sciences
- [ ] Ecological, evolutionary & environmental sciences

For a reference copy of the document with all sections, see nature.com/documents/nr-reporting-summary-flat.pdf

Life sciences study design

All studies must disclose on these points even when the disclosure is negative.

Sample size

Generally, the larger the sample size, the better of the statistical results of GWAS analysis will be. In order to obtain reliable and rational GWAS results, natural population sample size at least should be 200. In this study, male parental population and testcross population separately contained 113 inbred varieties and 565 (113×5) hybrid testcrosses. The phenotypes of a trait are denoted V for the inbred varieties and T for the testcross. Two additional types of observations were derived from V and T, including general combining ability (G) for inbred varieties and heterosis (H) for hybrid testcrosses. We developed a pipeline of joint phenotypes, effects, and generations (JPEG) and conducted three GWAS analyses with inbred varieties on two datasets (V and G, sample size is 113+113=226), hybrid testcrosses on two datasets (T and H, sample size is 565+565=1130), and their combination (V, G, T and H, sample size is 113+113+565+565 = 1356). The sample size of all of the three analysis were more than 200. In addition to that, the association was determined with the strict threshold of 1% type I error after Bonferroni multiple test correction on SNP markers. Both sample size and statistical methods well guaranteed the rationality of analysis results.

Data exclusions

In this study, phenotype and genotype of parental varieties (120) and hybrid testcrosses (575) were involved in analysis. For phenotype data, two male parental varieties (V2 and V3) and their hybrid testcrosses with missing phenotypes were excluded from the analyses. At last, 113 parental varieties and 565 (113×5) hybrid testcrosses were obtained for further GWAS analysis. For SNP genotype data, we first obtained a total of 7,734,465 raw SNPs from 120 parental varieties (115 male parental varieties and 5 female parental varieties) genotyped by whole genome sequencing with the Illumina HiSeq2500 platform, and then conducted quality control by deleting SNPs with a missing rate > 20% and minor allele frequency < 5%. In total, 1,619,588 SNPs were passed filters and quality control. Missing genotypes were imputed by NPUTE29 (version 4.0). Hybrid testcross genotypes were inferred using parental SNP genotypic information. This processing is a standard processing procedures of SNP genotype generating before GWAS analysis.

Replication

(1) The field trials of parental varieties and hybrid testcross lines were designed as randomized blocks and repeated twice. After the materials matured, three plants with uniform growth were selected from the middle eight plants, dried, and stored at room temperature for three months. 12 rice quality traits of male parental varieties and hybrid testcrosses were evaluated following the method of Lou et al. (QTL mapping of grain quality traits in rice. J. Cereal Sci. 50, 145–151 (2009).)

(2) A critical breeding goal is to identify new superior crosses with existing genotypes of inbred varieties and phenotypes of inbred varieties and their hybrids. The predictions for hybrids using the associated additive and dominant loci cannot be used to evaluate their predictability because of the overfitting that these loci were derived from all hybrids. To assess the capability, we reconducted GWAS with cross-validations. We randomly divided the hybrids into two groups. One group of hybrids was selected as a testing population. The remaining hybrid group and the parent inbred varieties were used as the training population. The testing population was iterated until all groups were tested. This process was repeated 100 times. The mean squared correlation coefficient across all groups and replicates was used to assess the capability to identify new superior crosses.

Randomization

The field trials of parental varieties and hybrid testcross lines were designed as randomized blocks and repeated twice. After the materials matured, three plants with uniform growth were selected from the middle eight plants, dried, and stored at room temperature for three months. 12 rice quality traits of male parental varieties and hybrid testcrosses were evaluated following the method of Lou et al. (QTL mapping of grain quality traits in rice. J. Cereal Sci. 50, 145–151 (2009).)

Blinding

A critical breeding goal is to identify new superior crosses with existing genotypes of inbred varieties and phenotypes of inbred varieties and their hybrids. The predictions for hybrids using the associated additive and dominant loci cannot be used to evaluate their predictability because of the overfitting that these loci were derived from all hybrids. To assess the capability, we reconducted GWAS with cross-validations. We randomly divided the hybrids into two groups. One group of hybrids was selected as a testing population. The remaining hybrid group and the parent inbred varieties were used as the training population. The testing population was iterated until all groups were tested. This process was repeated 100 times.

Reporting for specific materials, systems and methods

We require information from authors about some types of materials, experimental systems and methods used in many studies. Here, indicate whether each material, system or method listed is relevant to your study. If you are not sure if a list item applies to your research, read the appropriate section before selecting a response.

Materials & experimental systems

n/a	Involved in the study	
☒	☐	Antibodies
☒	☐	Eukaryotic cell lines
☒	☐	Palaeontology and archaeology
☒	☐	Animals and other organisms
☒	☐	Human research participants
☒	☐	Clinical data
☒	☐	Dual use research of concern

Methods

n/a	Involved in the study	
☒	☐	ChIP-seq
☒	☐	Flow cytometry
☒	☐	MRI-based neuroimaging
